# Enhanced Tumor Uptake and Retention of Cyanine Dye–Albumin Complex for Tumor-Targeted Imaging and Phototherapy

**DOI:** 10.3390/ijms24010862

**Published:** 2023-01-03

**Authors:** Gayoung Jo, Eun Jeong Kim, Hoon Hyun

**Affiliations:** 1 Department of Biomedical Sciences, Chonnam National University Medical School, Hwasun 58128, Republic of Korea; 2 BioMedical Sciences Graduate Program (BMSGP), Chonnam National University, Hwasun 58128, Republic of Korea

**Keywords:** IR-786, albumin, near-infrared fluorescence imaging, tumor targeting, photothermal therapy

## Abstract

Heptamethine cyanine dyes are widely used for in vivo near-infrared (NIR) fluorescence imaging and NIR laser-induced cancer phototherapy due to their good optical properties. Since most of heptamethine cyanine dyes available commercially are highly hydrophobic, they can usually be used for in vivo applications after formation of complexes with blood plasma proteins, especially serum albumin, to increase aqueous solubility. The complex formation between cyanine dyes and albumin improves the chemical stability and optical property of the hydrophobic cyanine dyes, which is the bottom of their practical use. In this study, the complexes between three different heptamethine cyanine dyes, namely clinically available indocyanine green (ICG), commercially available IR-786 and zwitterionic ZW800-Cl, and bovine serum albumin (BSA), were prepared to explore the effect of cyanine dyes on their tumor uptake and retention. Among the three complexes, IR-786©BSA exhibited increased tumor accumulation with prolonged tumor retention, compared to other complexes. Moreover, IR-786 bound to BSA played an important role in tumor growth suppression due to its cytotoxicity. To achieve complete tumor ablation, the tumor targeted by IR-786©BSA was further exposed to 808 nm laser irradiation for effective photothermal cancer treatment.

## 1. Introduction

Heptamethine cyanine dyes are typically applied in the near-infrared (NIR) fluorescence imaging of cancer after labeling with small molecules (e.g., drugs or ligands) and/or macromolecules (e.g., polymers or proteins). Several heptamethine cyanine dyes including IR-780, IR-783, IR-786, and IR-808 (also called MHI-148) are used solely for structure-inherent tumor targeting and antitumor activities without such preparation [1,2,3]. Moreover, the heptamethine cyanine dyes exhibit superior photothermal conversion capability under NIR light irradiation owing to their strong absorption in the NIR wavelength range of 650–950 nm. By combining the photothermal therapy (PTT) and chemotherapy, particularly due to their inherent cytotoxicity, the cyanine dyes armed with targeting abilities could provide efficient synergistic cancer therapy [4,5,6]. However, most of heptamethine cyanine dyes available commercially, including the FDA-approved NIR dye indocyanine green (ICG), show low water solubility, stability, and quantum yield, as well as a lack of tumor targeting specificity [7,8,9]. Although the commercial cyanine dyes are continuously employed for chemical conjugation to various small molecules (e.g., anticancer drugs or tumor targeting ligands), the target specificity of the conjugates may be altered depending on the cyanine dyes because of their unique physicochemical properties such as hydrophobicity, net surface charge, and polarity [6]. To be used for tumor-targeted imaging and phototherapy, the hydrophobic heptamethine cyanine dyes or their conjugates are either encapsulated or assembled into complex nanostructures [10,11,12,13,14].

Previously, organic anion transporting polypeptides (OATPs)-mediated uptake of the heptamethine cyanine dyes into cancer cells has been the predominant mechanism by which OATP transporters are well recognized to be overexpressed in a variety of cancer cells [15,16]. Recently, Usama et al. demonstrated that the tumor accumulation and persistence of the heptamethine cyanine dyes were mediated by the formation of noncovalent or covalent albumin adducts, which can be trapped in tumors via the receptor-mediated endocytosis of albumin [17,18]. Consequently, albumin is an ideal carrier protein with the advantages of biocompatibility, biodegradability, non-immunogenicity, and safety for the use of cyanine dyes in biomedical applications. The albumin–cyanine dye complexes, in which albumin serves as an active targeting carrier for the delivery of imaging agents to tumor sites, could be stable in systemic circulation to minimize off-target payload release after systemic administration [19,20]. Hence, the important role of albumin in carrying heptamethine cyanine dyes to the tumor tissue is to improve the water solubility and tumor targetability of the heptamethine cyanine dyes, thereby resulting in increased availability of such cyanine dyes.

Albumin is one of the most abundant proteins in the body and a transporter of various exogenous chemical compounds and molecules in the blood vascular system. Bovine serum albumin (BSA) is similar to the human serum albumin (HSA) with 80% sequence homology, and it has been widely used as a versatile carrier, due to its low cost, easy purification, and high loading capacity [21,22,23,24]. More importantly, BSA has both positively and negatively charged amino acids that allow the binding of both positively and negatively charged molecules, as well as both hydrophilic and hydrophobic compounds [21]. Because albumin preferentially accumulates at the tumor tissue, the clinically available heptamethine cyanine dye ICG has been successfully used for NIR fluorescence-guided tumor diagnosis and phototherapy after combining with HSA or BSA [10,25]. Additionally, it has previously been reported with other examples of different heptamethine cyanine dyes, such as hydrophobic IR-780 and hydrophilic IR-783, that their complexes with HSA or BSA showed not only improved blood circulation and tumor accumulation but also high photostability and fluorescence intensity suitable for NIR-II (1000–1700 nm) imaging [19,20]. Thus, albumin-chaperoned cyanine dye reveals enhanced water solubility, biocompatibility, stability, fluorescent brightness, and tumor targetability, satisfying the requirements for the clinical translation of cyanine dyes.

**Scheme 1:**
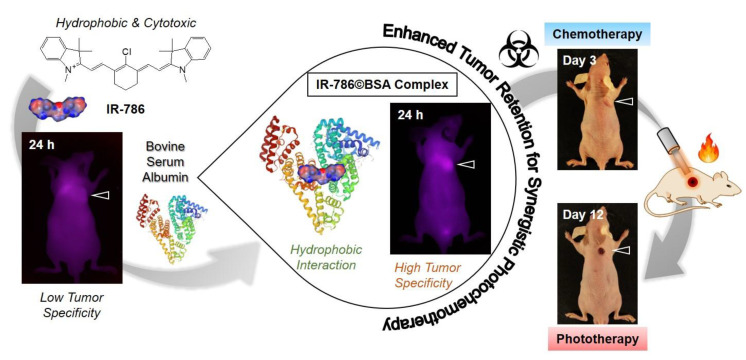
Schematic illustration of the complex formation between hydrophobic cyanine dye IR-786 and BSA for improved tumor imaging and synergistic photochemotherapy. The albumin-based complex provides a promising strategy for utilizing the hydrophobic and cytotoxic cyanine dyes for various biomedical applications.

In this study, we investigated the complexation behaviors of cyanine dyes and BSA using three different structures of heptamethine cyanine dyes, such as negatively charged ICG, positively charged IR-786, and zwitterionic ZW800-Cl. The clinically available ICG was used as a positive control, because the ICG©BSA complex has been studied previously [10,25]. Additionally, zwitterionic ZW800-Cl, well known as an analog of the charge-balanced ZW800-1, was used for identifying if the net surface charge distributed on the polymethine cyanine dye structure is a critical factor that affects the formation of complexes with BSA [26]. Importantly, the commercially available IR-786, known as highly hydrophobic and cytotoxic cyanine dye, was employed to form the complex with BSA and confirm whether the complex could also be useful for in vivo tumor imaging and therapy [27]. To the best of our knowledge, no study has investigated the phototherapeutic effect of the IR-786©BSA complex in tumor-bearing mice treated with NIR laser irradiation. The IR-786©BSA complex was further utilized as a tumor-targeted chemotherapeutic agent, due to the inherent cytotoxicity of IR-786, thereby enabling to effectuate synergistic photochemotherapy (Scheme 1). Therefore, this study provides a simple and effective strategy for the development of albumin–cyanine dye complexes to enhance the water stability, biocompatibility, and tumor targeting specificity of many different polymethine cyanine dyes for future clinical use.

## 2. Results

### 2.1. Preparation and Characterization of Cyanine Dye©BSA Complex

**Figure 1:**
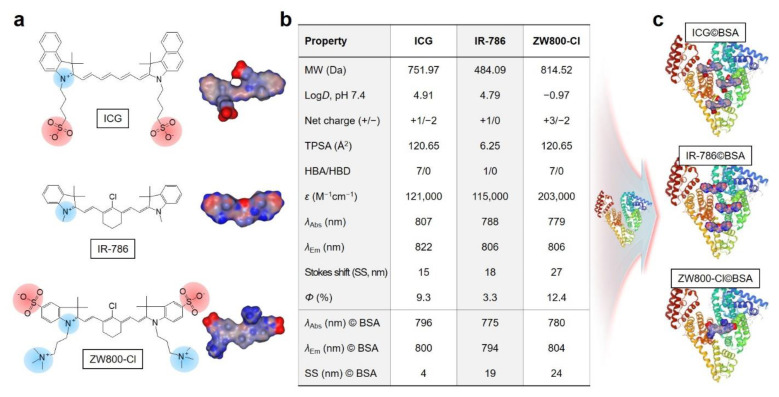
(**a**) Chemical structure, net surface charge, and three-dimensional (3D) modeling of the geometrical positions of ICG, IR-786, and ZW800-Cl NIR dyes. Red, negative charge; blue, positive charge; gray, hydrophobicity. (**b**) Physicochemical and optical properties of ICG, IR-786, ZW800-Cl in 100% serum, and their complexes with BSA in PBS at pH 7.4. In silico calculations of the distribution coefficient (log*D* at pH 7.4), topological polar surface area (TPSA), and hydrogen bond acceptors/donors (HBA/HBD) were performed using Marvin and JChem calculator plugins (ChemAxon). (**c**) Simplified schematic for the formation of each dye©BSA complex.

Chemical structures and three-dimensional modeling of the geometrical positions of ICG, IR-786, and ZW800-Cl cyanine dyes used in this study are shown in Figure 1a to compare the distribution coefficients for predictions of molecular surface charge and hydrophobicity. Among the various factors affecting biodistribution, in silico calculations of the physicochemical properties and optical properties such as absorbance and fluorescence emission maximum, and molar extinction coefficient of ICG, IR-786, and ZW800-1 are summarized in Figure 1b. In the present study, we selected the three different heptamethine cyanine dyes in terms of net surface charge, hydrophobicity, and possessing a chloro-cyclohexenyl ring on the heptamethine skeleton, which are highly important factors affecting the binding affinity with albumin. This approach involves the assembly of cyanine dyes with BSA through covalent or noncovalent (e.g., electrostatic, hydrophobic, and hydrogen-bonding) interactions to create a stable dye©BSA complex (Figure 1c).

**Figure 2:**
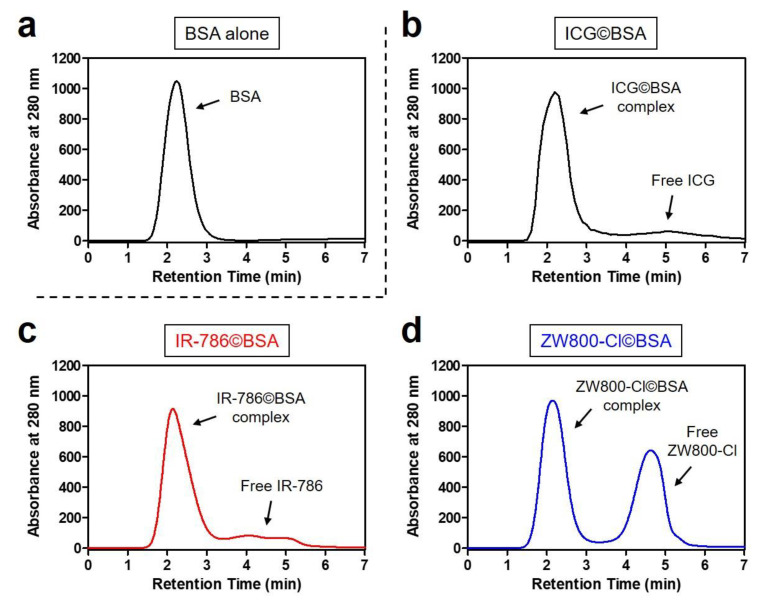
GFC separation of (**a**) BSA alone and its mixtures with (**b**) ICG, (**c**) IR-786, and (**d**) ZW800-Cl. The mixture was incubated at 37 °C for 4 h prior to loading on a separating column. The hydrodynamic diameter of BSA complex was measured at 280 nm using an absorbance spectrophotometer.

The mixtures of cyanine dye©BSA complexes were uniformly purified by a gel-filtration chromatography (GFC) system to eliminate the free dye residue that would affect in vivo NIR fluorescence imaging. After confirming the retention time of BSA alone, the dye©BSA complexes can be identified by their different retention times between BSA complex and unbound dye (Figure 2a). Interestingly, ICG©BSA and IR-786©BSA complexes showed similar patterns of free dye separation, whereas ZW800-Cl@BSA revealed a large amount of unbound ZW800-Cl (Figure 2b–d). Although the low binding affinity of ZW800-Cl to BSA is still unclear, the zwitterionic property and high hydrophilicity of ZW800-Cl may contribute to the prevention of covalent binding and hydrophobic interaction with BSA.

**Figure 3:**
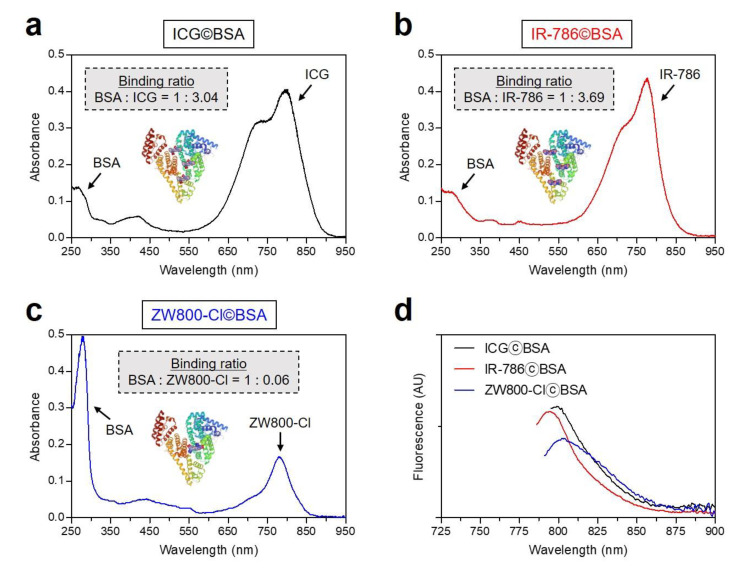
Absorption spectra of (**a**) ICG©BSA, (**b**) IR-786©BSA, and (**c**) ZW800-Cl©BSA complexes, and (**d**) their fluorescence emission spectra. The sample was obtained from the 1st peak fraction as shown in Figure 2. Optical measurements were performed in PBS, pH 7.4. The binding ratio of each dye©BSA complex was calculated by the molar extinction coefficient and absorbance peak of each dye and BSA, as follows: Binding ratio = (Abs_dye max_/*ε*_dye_)/[(Abs_280 nm_ − CF × Abs_dye max_)/*ε*_BSA_], CF = correction factor: ICG = 0.02, IR-786 = 0.04, ZW800-Cl = 0.02, ε_BSA_ = 43,824 M^−1^ cm^−1^.

To compare the binding ratios between cyanine dyes and BSA, the binding ratio of each dye©BSA complex was calculated by the molar extinction coefficient and absorbance peak of each dye and BSA, based on the equation reported previously [28,29]. The binding ratios of ICG©BSA and IR-786©BSA complexes were determined as BSA-to-dye ratios of 3.04 and 3.69, respectively (Figure 3a,b). However, the ZW800-Cl©BSA complex has an extremely low binding ratio of 0.06, which is consistent with the result of GFC separation (Figure 3c). This indicates that the hydrophobic ICG and IR-786 dyes can effectively bind to BSA by hydrophobic interactions, unlike the hydrophilic zwitterionic ZW800-Cl. Interestingly, the absorbance and fluorescence emission maximum of ICG©BSA and IR-786©BSA complexes showed the hypochromic shifts compared to that of dye alone, except for ZW800-Cl©BSA (Figure 1b and Figure 3d). Due to a change in environmental conditions, we presume that the improved water solubilities of ICG and IR-786 dyes may contribute to their optical properties.

### 2.2. In Vitro Cytotoxicity and In Vivo Tumor Targeting of Cyanine Dyes

**Figure 4:**
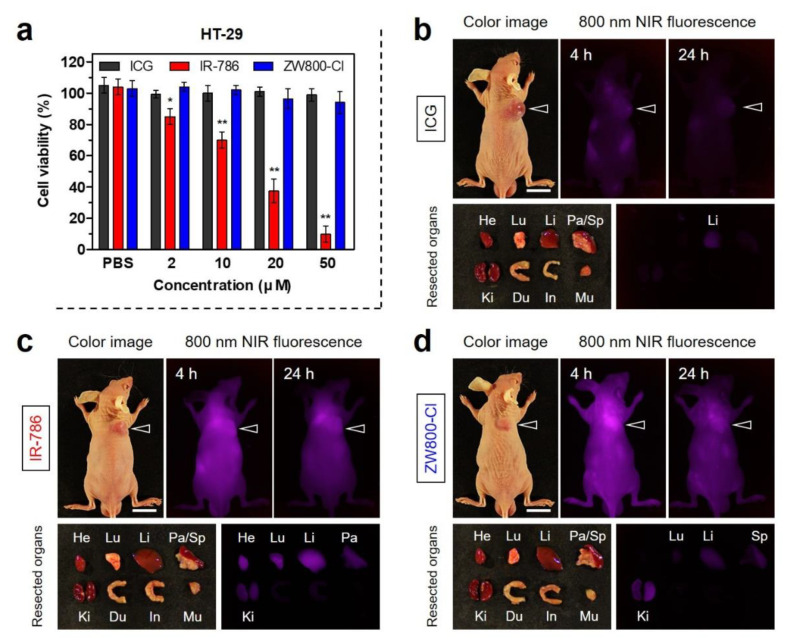
(**a**) Cell viability assay of each NIR dye using HT-29 cancer cells. The percentage cytotoxicity was determined after 24 h of treatment with various concentrations of each NIR dye. Data are expressed as mean ± S.D. (*n* = 3, * *p* < 0.05; ** *p* < 0.01). In vivo HT-29 tumor targeting efficiency and biodistribution of (**b**) ICG, (**c**) IR-786, and (**d**) ZW800-Cl. The tumor site and resected major organs were imaged 24 h after injection of each NIR dye, respectively. The tumor sites are indicated by arrowheads. Abbreviations: Du, duodenum; He, heart; In, intestines; Ki, kidneys; Li, liver; Lu, lungs; Mu, muscle; Pa, pancreas; Sp, spleen. Scale bars = 1 cm. Images are representative of 3 mice per treatment group. All NIR fluorescence images had identical exposure times and normalization.

The in vitro cytotoxicity test was conducted using the HT-29 cancer cell line, because the HT-29 xenograft tumor model was used for in vivo studies. To determine the cell viability, the MTT assay was performed in HT-29 cancer cells after incubation with various concentrations of ICG, IR-786, and ZW800-Cl for 24 h (Figure 4a). As expected, no significant cytotoxicity of ICG and ZW800-Cl was observed in the concentration range of 2–50 μM. It is well known that ICG is the FDA-approved clinical dye and ZW800-Cl is an analog of the biocompatible zwitterionic NIR dye ZW800-1, as reported previously [7,26]. However, IR-786 displayed considerable cytotoxicity with an increase in concentration, which is consistent with the previous study [27,30]. Based on the concentration-dependent cytotoxicity of IR-786, the half maximal inhibitory concentration (IC50) was determined to be 14.9 μM. This indicates that IR-786 may be used as a chemotherapeutic agent depending on its preferential accumulation in the tumor. Subsequently, the in vivo tumor targeting of each dye before combining with BSA was investigated in the HT-29 xenograft tumor model. ICG, IR-786, and ZW800-Cl dyes were intravenously injected into tumor-bearing mice at 24 h prior to imaging. The time-dependent NIR fluorescence imaging showed no significant fluorescence signals from the tumor sites injected with ICG and IR-786 for 24 h post-injection (Figure 4b,c). As expected, ICG was mostly eliminated by hepatobiliary excretion with little uptake in other organs within 24 h post-injection, while IR-786 displayed high nonspecific uptake in major organs including heart, lungs, liver, pancreas, and kidneys at 24 h post-injection. Unlike them, ZW800-Cl showed significant uptake in the tumor within 4 h of injection, then the fluorescence signal at the tumor site gradually decreased until 24 h post-injection (Figure 4d). The tumor targetability and biodistribution of ICG, IR-786, and ZW800-Cl dyes are consistent with the previous reports [9,26,27]. In this regard, the complexation between BSA and such cyanine dyes is required to improve tumor accumulation and retention for tumor-targeted imaging and phototherapy.

### 2.3. Time-Dependent In Vivo Tumor Retention and Chemotherapeutic Efficacy

**Figure 5:**
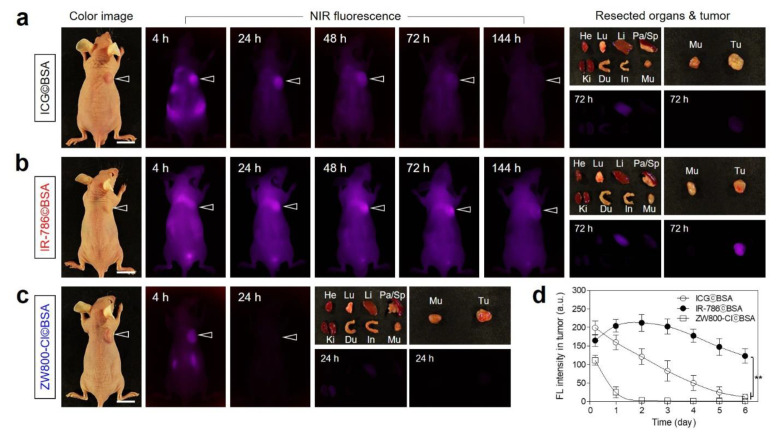
In vivo HT-29 tumor targeting efficiency and biodistribution of (**a**) ICG©BSA, (**b**) IR-786©BSA, and (**c**) ZW800-Cl©BSA complexes. (**d**) Time-dependent fluorescence intensities at the tumor sites targeted by dye©BSA complexes. The tumor site and resected major organs were imaged after injection of each dye©BSA complex during a designated period of time, respectively. The tumor sites are indicated by arrowheads. Abbreviations: Du, duodenum; He, heart; In, intestines; Ki, kidneys; Li, liver; Lu, lungs; Mu, muscle; Pa, pancreas; Sp, spleen; Tu, tumor. Scale bars = 1 cm. Images are representative of 3 mice per treatment group. All NIR fluorescence images had identical exposure times and normalization. Data are expressed as mean ± S.D. (*n* = 3, ** *p* < 0.01).

After confirming the tumor targetability and biodistribution of the cyanine dye itself, the tumor-specific targeting of cyanine dye©BSA complexes was reconfirmed in the HT-29 xenograft tumor model. Each cyanine dye©BSA complex was injected intravenously into the tumor-bearing mice, subsequently, real-time NIR fluorescence imaging was continually performed for 6 days. Although the high fluorescence signal from the tumor tissue injected with ICG©BSA was observed at 4 h post-injection, the fluorescence intensity targeted in the tumor tissue was continuously diminished and finally undetectable at 6 days post-injection (Figure 5a,d). This demonstrates that the noncovalent binding between ICG and BSA, due to the absence of the chloro-cyclohexenyl ring in the chemical structure of ICG, is relatively weak binding and allows ICG escape from BSA after tumor accumulation. Unlike ICG©BSA, the fluorescence intensity at the tumor site treated with IR-786©BSA dramatically increased for 2 days after administration and gradually decreased during the next 4 days (Figure 5b,d). This indicates that the chloride-containing IR-786 could be stably bound to the BSA through covalent bonding, thereby resulting in prolonged tumor retention, compared to that of ICG©BSA. Interestingly, ZW800-Cl©BSA showed poor tumor uptake even at 4 h post-injection and the fluorescence signal in the tumor tissue was almost undetectable at 24 h post-injection (Figure 5c,d). The weak fluorescence intensity at the tumor site corresponded to the low binding ratio of ZW800-Cl to BSA. Although ZW800-Cl is a heptamethine cyanine dye consisting of the chloro-cyclohexenyl ring capable of binding to BSA covalently, the zwitterionic surface charge may contribute to the prevention of covalent binding between ZW800-Cl and BSA.

**Figure 6:**
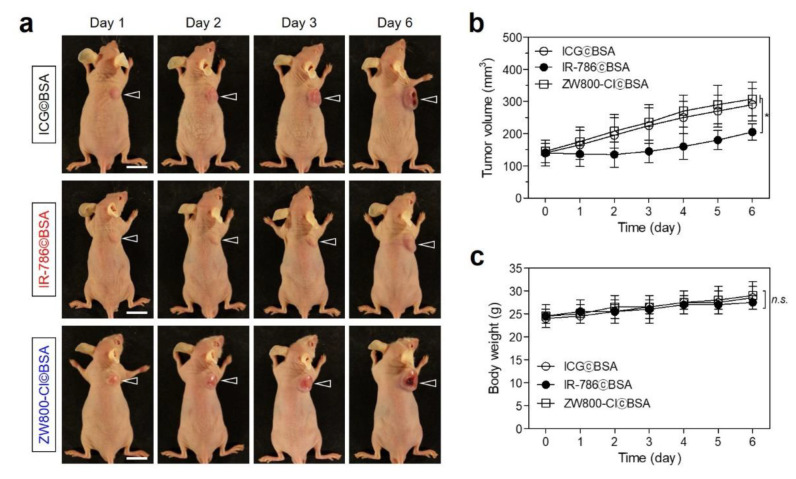
In vivo chemotherapeutic efficacy. (**a**) Representative photos of tumor size changes in HT-29 tumor-bearing mice for 6 days after different treatments. The tumor sites are indicated by arrowheads. Scale bars = 1 cm. (**b**) Tumor growth rates and (**c**) body weights of each treatment group were monitored for 6 days. Images are representative of 3 mice per treatment group. Data are expressed as mean ± S.D. (*n* = 3, * *p* < 0.05; *n.s.* = not significant).

To evaluate the chemotherapeutic effect of IR-786©BSA, HT-29 tumor-bearing mice were continuously monitored after a single-dose injection without additional treatments (Figure 6a). The tumor growth in each treatment group was observed for 6 days to confirm the tumor-suppressive function of the cyanine dyes as shown by the in vitro cytotoxicity test. As expected, the treatment groups of ICG©BSA and ZW800-Cl©BSA showed no therapeutic effects with similar growth rates at their tumor sites. Importantly, the tumors in the IR-786©BSA treatment group revealed delayed growth during the early stage of treatment for 3 days, but the tumor volumes gradually increased during the next 3 days (Figure 6b). This indicates that the IR-786 could contribute to the tumor suppression for a certain period of time due to its high cytotoxicity compared to that of ICG and ZW800-Cl. Moreover, the BSA as an active targeting carrier played a key role in site-specific delivery and prolonged retention of IR-786 at the tumor site. Since the IR-786©BSA complex still remained in the tumor tissue at 3 days post-injection with sufficient fluorescence intensity, IR-786©BSA is also capable of the photothermal cancer treatment to destruct entire tumors. Additionally, the body weights of the mice in each treatment group were measured during their monitoring period (Figure 6c). The IR-786©BSA treatment group displayed no significant change in body weight, similar to other groups, which suggests a favorable tolerance of the chemotherapy and improved biosafety of the IR-786©BSA complex.

### 2.4. In Vivo Photothermal Therapeutic Efficacy

**Figure 7:**
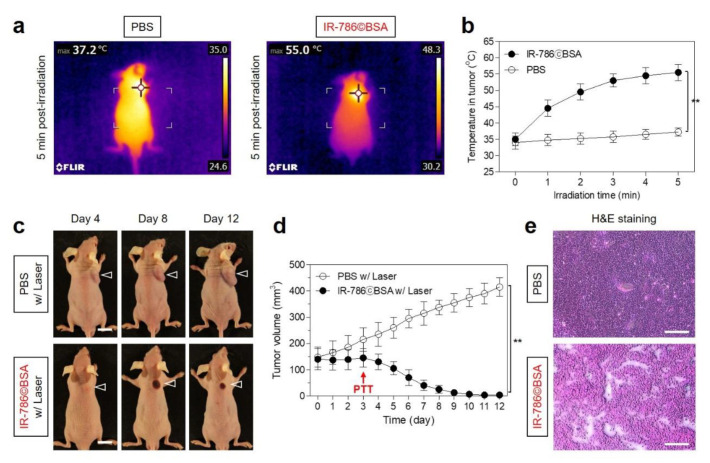
In vivo NIR phototherapeutic efficacy. (**a**) Whole-body thermal images of tumor-bearing mice 72 h after injection of PBS or IR-786©BSA upon exposure to 808 nm laser irradiation (1.1 W/cm^2^) for 5 min. (**b**) Temperature changes at the tumor sites in each treatment group were monitored during the 5 min of 808 nm laser irradiation. (**c**) Representative photos of tumor size changes in HT-29 tumor-bearing mice for 12 days after different treatments. The tumor sites are indicated by arrowheads. Scale bars = 1 cm. Images are representative of 3 mice per treatment group. (**d**) Tumor growth rates of each treatment group were monitored for 12 days. (**e**) Tumor sections stained with H&E from each group after 24 h of different treatments. Scale bars = 100 μm. Data are expressed as mean ± S.D. (*n* = 3, ** *p* < 0.01).

After confirming the certain chemotherapeutic effect and sustained tumor accumulation of IR-786©BSA up to 3 days after injection, the PTT capability study of the IR-786©BSA complex in vivo was conducted in the HT-29 xenograft tumor model under NIR laser irradiation. IR-786©BSA or PBS alone were intravenously injected into the tumor-bearing mice 72 h before laser irradiation. Subsequently, the tumor was exposed to 808 nm NIR laser irradiation at 1.1 W/cm^2^ for 5 min. The power density of the NIR laser was optimized to prevent normal tissue damage occurring by the laser power itself without PTT agents, as reported previously [31,32]. The temperature of the tumors treated with IR-786©BSA 72 h post-injection significantly increased up to 55 °C during the 5 min of laser irradiation, whereas tumor temperatures in the PBS-injected mice showed little change (37.2 °C) under the same condition (Figure 7a). This indicates that the IR-786©BSA complex remained in the tumor tissue at 72 h after injection was good enough to generate photothermal energy for cancer ablation therapy. The tumors treated with IR-786©BSA exhibited a phototherapeutic temperature close to 50 °C for 2 min after irradiation, then the tumor temperature was maintained up to 55 °C for the next 3 min of laser irradiation (Figure 7b). The increased tumor temperature generated by IR-786©BSA is sufficient to induce tumor necrosis; therefore, IR-786©BSA can be used for tumor-targeted imaging, prolonged tumor retention, and effective photothermal cancer therapy. Finally, the in vivo phototherapeutic efficacy of IR-786©BSA was monitored for 12 days after PTT treatment (Figure 7c,d). The PBS treatment group showed no skin damage (e.g., burn scarring) and tumor suppression induced by the laser power itself, while tumor tissues in the IR-786©BSA-injected mice revealed a notable PTT effect with complete tumor ablation and no recurrence during the course of the treatment. In addition, the PTT effect was reconfirmed by the H&E staining of the tumor tissues obtained from each group 24 h after laser irradiation (Figure 7e). The tumor sections treated with IR-786©BSA and laser irradiation showed complete necrosis with shrunken nuclei, whereas no change in the cancer cells was observed in the tumor sections treated with PBS and laser irradiation. This demonstrates that the IR-786©BSA complex can be successfully used as an effective PTT agent for complete tumor ablation.

## 3. Discussion

Tumor-targeted imaging using exogenous contrast agents is a very important tool for the successful cancer diagnosis and surgical treatment. Particularly, a number of NIR fluorescent dyes have been utilized to monitor biological processes and the diagnosis of diseases after conjugation with small molecule drugs or antibodies. The development of NIR fluorescent cyanine dyes enabling target-specific imaging is of great importance because they can provide the real-time information on where, when, and how fluorescent conjugates are accumulated, activated, and cleared in the body. However, the clinical use of the commercial cyanine dyes is still limited due to their low water stability, high nonspecific tissue/organ uptake, and potential long-term toxicity. Although the ICG is currently the only clinically used NIR dye, free ICG typically showed short plasma half-life, poor water stability, and a lack of tumor specificity [7,8,9]. Hence, the complex of ICG with serum albumin has been increasingly used to improve the blood circulation, photostability, and tumor targeting ability of ICG for clinical and biomedical applications [10,25]. Moreover, the ICG/BSA complex was successfully applied to photothermal therapy after tumor-targeted accumulation [10,33]. The albumin is a promising carrier for targeted tumor imaging and drug delivery because it can be selectively accumulated inside the tumor interstitium, known as the enhanced permeation and retention (EPR) effect [21]. Additionally, the tumor-specific uptake of albumin can also be explained by receptor-mediated albumin uptake pathways involved with albumin binding proteins such as membrane-associated glycoprotein and secreted protein acidic and rich in cysteine (SPARC) [21]. Thus, the exogenous albumin can serve as a versatile carrier for prolonged blood circulation and enhanced tumor accumulation of commercial cyanine dyes, enabling long-term bioimaging and NIR laser-activated cancer phototherapy.

Here, we show that binding IR-786 to BSA allows it to preferentially accumulate in HT-29 xenograft tumors, where it can prolong tumor retention, leading to reduced tumor growth and complete tumor ablation combined with PTT. It is noteworthy that IR-786©BSA has enhanced tumor targeting ability in tumor-bearing mice, compared to that of ICG©BSA. Although the prolonged tumor retention of IR-786©BSA is still unclear, it was explained that heptamethine cyanine dyes with a meso-chloride (i.e., IR-780 and IR-783) tend to be much longer living in tumors than heptamethine cyanine dyes without a meso-chloride (i.e., ICG), owing to the difference in covalent or noncovalent binding to albumin [17]. Consequently, the cytotoxic IR-786 could contribute to the tumor suppression during the accumulation period after release from BSA. Previously, Usama et al. demonstrated that the chloride-containing cyanine dyes could be transformed into covalent adducts with serum proteins possessing free thiols, after an injection into the blood, thereby resulting in persistent fluorescence from tumor tissue in vivo [17,18]. In addition, they suggested that various factors might contribute to the persistent localization of covalent adducts formed in situ, including the slow efflux and the EPR effect. Therefore, IR-786 showed a much more favorable binding with albumin than that of ICG, due to the presence of the chloro-cyclohexenyl ring.

Although the binding mechanism between chloride-containing cyanine dyes and albumin is reasonable for the long-term uptake and persistent tumor localization, the chloride-containing cyanine dyes with different structures (i.e., IR-786 and ZW800-Cl) revealed entirely different binding affinities to albumin. It is well known that the zwitterionic NIR dye (i.e., ZW800-1) bearing balanced charge groups has low serum binding, low cellular permeability, and ultralow nonspecific uptake in vivo. In this regard, this suggests that the zwitterionic property of ZW800-Cl may contribute to the prevention of covalent binding between the chloro-cyclohexenyl ring of ZW800-Cl and the free thiol of BSA. Moreover, we presume that the high hydrophilicity of ZW800-Cl may influence the less binding interaction with the hydrophobic cavity of albumin, compared to that of the hydrophobic dye IR-786, which is consistent with the previous study [20,34]. Based on the crystal structure of albumin, BSA has two ligand binding sites (designated as Site I and Site II). The binding Site I has strong hydrophobic interactions because of the presence of 16 hydrophobic residues, whereas the binding Site II involves not only hydrophobic interaction but also hydrogen bonding and electrostatic interactions [34]. Furthermore, Bai et al. clarified that the general principle is to design either albumin-chaperoned or albumin-escaping dyes by using hydrophobic IR-780 and hydrophilic IR-783 dyes containing the chloro-cyclohexenyl ring. The authors suggested that the slow binding between cyanine dye and albumin is considered as a noncovalent combination and the cyanine dye could be a potential albumin-escaping agent [20].

In summary, we demonstrated how the physicochemical and molecular properties of heptamethine cyanine dyes influence in albumin binding of cyanine dyes to achieve better cancer treatment performance. The IR-786©BSA complex appears to be a potential agent for photochemotherapy that, when combined with the NIR laser irradiation, can lead to an efficient synergistic cancer therapy. This study provides a simple and effective approach to utilizing the many different polymethine cyanine dyes, possessing high hydrophobicity and cytotoxicity, for various biological applications.

## 4. Materials and Methods

### 4.1. Preparation of Cyanine Dye©BSA Complex

All reagents and solvents were purchased from Sigma-Aldrich (St. Louis, MO, USA) and were used without further purification. The ZW800-Cl heptamethine cyanine dye was prepared as described previously [35,36]. A mixture of bovine serum albumin (BSA, MW ≈ 67 kDa; 0.15 μmol, 10 mg/mL, 150 μM) and cyanine dyes (ICG, IR-786, and ZW800-Cl; 0.5 μmol, 500 μM) in phosphate buffered saline (PBS; 1 mL, pH 7.4) was incubated at 37 °C for 4 h, respectively. The mixture was separated using a gel-filtration chromatography (GFC) system with Econo-Pac P6 cartridges (Bio-Rad, Hercules, CA, USA) and a flow rate of 1 mL/min (PBS, pH 7.4). The hydrodynamic diameter of BSA complex was measured at 280 nm using the ÄKTA start™ system (GE Healthcare, Piscataway, NJ, USA). The separated dye©BSA complexes were analyzed using a fiber optic UV-Vis-NIR (200–1025 nm) spectrometer (Ocean Optics, Dunedin, FL, USA).

### 4.2. Optical and Physicochemical Property Analyses

Optical properties of dye©BSA complexes were measured in PBS at pH 7.4. The absorption spectra of dye©BSA complexes were measured by an absorbance spectrometer (Ocean Optics). The molar extinction coefficient (*ε*) of each dye was calculated using the Beer–Lambert equation. The fluorescence emission spectra of dye©BSA complexes were recorded using a SPARK^®®^ 10 M microplate reader (Tecan, Männedorf, Switzerland) at an excitation wavelength of 750 nm and emission wavelengths ranging from 770 to 900 nm. In silico predictions of the distribution coefficient (log*D* at pH 7.4), topological polar surface area (TPSA), surface molecular charge, and hydrogen bond acceptors/donors (HBA/HBD) were performed using Marvin and JChem calculator plugins (ChemAxon, Budapest, Hungary).

### 4.3. In Vitro Cytotoxicity Assay

The human colorectal adenocarcinoma cell line HT-29 was obtained from the American Type Culture Collection (ATCC, Manassas, VA, USA). Cancer cells were maintained in Roswell Park Memorial Institute (RPMI) 1640 medium (Gibco BRL, Paisley, UK) supplemented with a 10% fetal bovine serum (FBS, Gibco BRL) and an antibiotic–antimycotic solution (Welgene, Daegu, South Korea) in a humidified 5% CO_2_ atmosphere at 37 °C. When the cells reached a confluence of approximately 50%, cell toxicity and proliferation were evaluated using a 3-(4,5-dimethylthiazol-2-yl)-2,5-diphenyltetrazolium bromide (MTT, Sigma-Aldrich) assay. The HT-29 cells were seeded onto 96-well plates (1 × 10^4^ cells per well). To evaluate the cytotoxicity depending on the dye concentration, the cancer cells were treated with each dye (2, 10, 20, and 50 μM) for 1 h and cultured for 24 h after treatment. At each time point, the incubation cell medium was replaced with 100 μL of fresh medium, and 10 μL of the MTT solution was directly added to each 100 μL well. Subsequently, the plates were then incubated for 4 h at 37 °C in a humidified 5% CO_2_ incubator. Finally, the plates were placed in a microplate reader (SPARK^®®^ 10 M, Tecan) to measure the absorption intensity at 570 nm. Cell viability was calculated using the following formula: cell viability (%) = (*A*_sample_ − *A*_blank_)/(*A*_control_ − *A*_blank_) × 100, where *A* is the average absorbance.

### 4.4. HT-29 Xenograft Mouse Model

Animal studies were performed in accordance with the guidelines approved by the Chonnam National University Animal Research Committee (CNU IACUC-H-2020-19). Adult (6-week-old, ≈25 g) male athymic nude mice were purchased from OrientBio (Gwangju, South Korea). HT-29 cancer cells were cultured and suspended in 100 μL of PBS before being subcutaneously inoculated in the right flank of each mouse (1 × 10^6^ cells per mouse). When tumor sizes reached about 1 cm in diameter between 8 and 10 days post-inoculation, each dye©BSA complex was administered intravenously. Animals were euthanized for in vivo NIR fluorescence imaging within a designated period of time.

### 4.5. In Vivo Biodistribution and Tumor Imaging

In vivo NIR fluorescence imaging was performed using an FOBI imaging system (NeoScience, Suwon, Republic of Korea). Mice (3 mice per treatment group) were sacrificed 24 or 72 h after injection, and their main organs (heart, lungs, liver, pancreas, spleen, kidneys, duodenum, and intestine) were harvested and imaged to confirm the time-dependent biodistribution of dye©BSA complexes. The fluorescence intensities of the tumors and excised organs were analyzed using ImageJ software (National Institutes of Health, Bethesda, MD, USA).

### 4.6. In Vivo Photothermal Therapeutic Efficacy

IR-786©BSA or PBS were intravenously injected into the HT-29 tumor-bearing mice (3 mice per treatment group) and the mice were anaesthetized after 72 h. The tumors were treated with a laser (1.1 W/cm^2^, *λ* = 808 nm) for 5 min. Temperature changes at the tumor sites were monitored using a thermal imager (FLIR Systems, Wilsonville, OR, USA). Tumors were excised from the treated mice 24 h after irradiation for subsequent analysis of histological samples stained with hematoxylin and eosin (H&E). To assess the in vivo antitumor effect, the macroscopic tumor growth of each group was observed for 12 days. The tumor volume (V) was measured by the following formula: V = 0.5 × longest diameter × (shortest diameter)^2^.

### 4.7. Statistical Analysis

Statistical analysis was performed by one-way analysis of variance for multiple comparison test. The results were represented as mean ± standard deviation (S.D.). A value of *p* < 0.05 was considered statistically significant. Curve fitting was performed using the Prism software (GraphPad, San Diego, CA, USA).

### 4.8. Histological Analysis

Resected tumors were preserved for H&E staining and microscopic observation. The tumors were fixed in 4% paraformaldehyde and flash-frozen in an optimal cutting temperature (OCT) compound using liquid nitrogen. Frozen samples were cryosectioned (10 µm thick), stained with H&E, and observed using a microscope. Histological analysis was performed on a Nikon Eclipse Ti-U inverted microscope system (Nikon, Seoul, South Korea). Image acquisition and analysis were performed using the NIS-Elements Basic Research software (Nikon).

## Data Availability

Not applicable.
